# Compliance and approach to voluntary HIV testing in a high-risk region for HIV transmission in Europe

**DOI:** 10.4314/ahs.v22i4.55

**Published:** 2022-12

**Authors:** Marija Milic, Jelena Dotlic, Jasmina Stevanovic, Katarina Mitic, Desmond Nicholson, Andrijana Karanovic, Amila Vujacic, Tatjana Gazibara

**Affiliations:** 1 Department of Epidemiology, Faculty of Medicine, University of Pristina temporarily seated in Kosovska Mitrovica, Kosovska Mitrovica, Kosovo, Serbia; 2 Clinic for Obstetrics and Gynecology, Clinical Center of Serbia, Faculty of Medicine, University of Belgrade, Belgrade, Serbia; 3 Department of Epidemiology, Faculty of Medicine, University of Pristina temporarily seated in Kosovska Mitrovica, Kosovska Mitrovica, Kosovo, Serbia; 4 Program Coordinator of the Emergency Relief Project “Solidarity”, SOS Children's Villages Serbia; 5 Department of Regional Health Services Region Five, Ministry of Public Health, Georgetown, Guyana; 6 Department of Microbiology, Faculty of Medicine, University of Pristina temporarily seated in Kosovska Mitrovica, Kosovska Mitrovica, Kosovo, Serbia; 7 School of Dentistry, University of Pristina temporarily seated in Kosovska Mitrovica, Kosovska Mitrovica, Kosovo, Serbia; 8 Institute of Epidemiology, Faculty of Medicine, University of Belgrade, Belgrade, Serbia

**Keywords:** HIV testing, attitude, knowledge, University students

## Abstract

**Background:**

The Kosovo province is being considered as a high-risk region for the spread of HIV.

**Objective:**

To estimate the prevalence and factors associated with HIV testing in a sample of university students from the Serbian northern Kosovo province.

**Material and Methods:**

A questionnaire examining socio-demographic characteristics, HIV-related knowledge, attitudes towards people living with HIV (PLHIV) and HIV testing was used in data collection. A total of 1,017 students from the University of Priština temporarily seated in Kosovska Mitrovica completed the questionnaire.

**Results:**

Only 5.4% of students have previously been tested for HIV, even though the majority (70.9%) had a positive approach to HIV testing. Factors associated with having been tested for HIV were being male and younger, having interest in HIV testing and having previous contact with PLHIV. Being more knowledgeable about HIV and having stronger positive attitude towards PLHIV, being older, receiving information about HIV through friends and special educational programs, using condom at last sexual intercourse, having positive opinion on gays/lesbians and previous contact with PLHIV were associated with positive approach to HIV testing.

**Conclusion:**

Having a positive approach to HIV testing does not suggest that students would take the HIV test. However, students who have low HIV-related knowledge, negative approach or lack of interest in HIV testing (believing that there is no need to take it) would likely never take the HIV test. Increasing HIV-related knowledge, acceptance of PLHIV and access to testing facilities should be public health priorities to raise HIV testing rates.

## Introduction

Only 2.5% of the population aged 15–49 years (1.4% ≤24 years) in Serbia had ever been tested for HIV 1. The rate of voluntary (i.e., client-initiated) HIV counselling and testing is almost five times lower than that initiated by health care providers 2. Moreover, during the past decade, the HIV testing frequency in Serbia has declined, even though an apparent increase in the HIV incidence was reported 3–5. By the end of 2019, there were 2,780 people living with HIV (PLHIV) in the Republic of Serbia and the estimated prevalence in the population aged above 15 was less than 0.1% 5. The highest age-specific HIV incidence rate was registered among people aged 30–39 years (7.99 / 100,000) and 20–29 years (6.38 / 100,000)^5^. Unprotected sexual contact was registered as the most common mode of transmission of HIV in Serbia, and 11 infected men were registered per one infected woman 5. Because timely testing facilitates the initiation of adequate treatment of HIV, the low testing rate presents a major obstacle to the effective control of the HIV spread^6^–^8^.

The Serbian province of Kosovo has had numerous socio-economic, educational and health challenges as a result of previous armed conflict between the Serbian (predominantly living in North Kosovo) and the Albanian community (predominantly living in the remainder of Kosovo). While the scale of the HIV epidemic in Kosovo is deemed small, the prevalence of new HIV diagnoses in high-risk groups (men who have sex with men, injecting drug users and sex workers) is five times higher than that in the general population 9. Factors such as the overall young adult population, limited business opportunities and high rates of unemployment, ongoing ethnic tensions, migrations, availability of inexpensive illicit substances and alcohol as well as developed sex industry due to the presence of foreign military and non-governmental organizations facilitate the HIV spread 5,10. Finally, the only counselling center for sexually transmitted diseases (STDs) and HIV available in this area has low visitation rates 11. As a result, the Kosovo province is being considered as a high-risk region for the spread of STDs including HIV 5,10.

The aims of this study were to: 1) estimate the prevalence of HIV testing in a sample of university students from the Northern Kosovo province; 2) examine factors associated with compliance and positive approach to HIV testing.

## Methods

### Setting

The study was carried out from February 1 to June 1, 2014 at the University of Pristina temporarily seated in Kosovska Mitrovica, Northern Kosovo province. Kosovska Mitrovica has approximately 30,000 Serbian ethnicity residents. The University of Pristina temporarily seated in Kosovska Mitrovica is the only educational institution in Kosovo that provides high education in Serbian language. The University is composed of 10 schools with more than 5,000 undergraduate students. This setup allows for a unique opportunity to study university student population in the Serbian Kosovo province.

### Participants

Students in the first and fourth study year were enrolled. Such selection enabled the recognition of different approaches to voluntary HIV testing at the beginning and at the end of university schooling. It also presented an indirect impact of education and growing up on testing approach over time. Participants were approached in all available classrooms during mandatory lessons on two randomly selected working days (Mondays and Thursdays). All ten schools were listed alphabetically and study recruitment lasted for one week in each school.

The principal investigator (PI, the first author of this paper) in this research made contact with all the schools to arrange for the time of questionnaire distribution. The questionnaires were distributed after the compulsory classes have ended, while students were still in the classroom. The PI provided a detailed explanation about the questionnaire and the study purpose, highlighting that the survey was voluntary and anonymous. In case of refusal to complete the questionnaire, the students were allowed to leave the classroom and no coercion was in place. The PI was at students' disposal for any questions related to the study topic, survey and questionnaire. The informed consent was provided before the paper questionnaire was handed over to the potential participant by the PI inside the classrooms.

Out of the invited 1,225 students, the response rate was 82.0%. The Ethics Committee of the Faculty of Medicine, University of Pristina temporally seated in Kosovska Mitrovica approved the study (No. 09-1608-1, issued on October 29, 2013).

### Instrument

We distributed a questionnaire, developed within the 8th round of the Global Fund for the Fight against AIDS, Tuberculosis and Malaria of the European Union (GFATM) project (UNAIDS) and then modified for the Serbian setting during the project “Strengthening HIV Prevention and Care for the Groups Most Vulnerable to HIV/AIDS” (Grant no. SER-809-G04-H and SER-809-G05-H; project funder the Global Fund for the Fight against AIDS, Tuberculosis and Malaria) 12. Questions about students' lifestyle were added based on data from literature suggesting that young people are more likely to engage in risky behaviours because of sensation-seeking^13^. The final version of the questionnaire had 58 items (Appendix). The questionnaire was tested in a pilot study after which the acceptability and clarity of the questionnaire were confirmed suggesting that the study was feasible.

The questionnaire explored participants' socio-demographic characteristics (six items), sources of information about HIV infection (six items), knowledge about HIV infection and transmission (14 items), previous HIV testing experiences, awareness and interest in HIV testing and self-perception of HIV risk (five items), attitudes towards PLHIV (17 items), opinions about high-risk groups for HIV (gays/lesbians, injecting drug users and sex workers), having had STDs in the past year and practice of risky behaviours (seven items). Only one answer per question was previewed.

Responses to items about HIV-related knowledge were scored as follows: one point for an incorrect answer; two points for not knowing or not being sure about a correct answer; and three points for a correct answer. The raw continuous Knowledge Score (KNS) was calculated as the sum of points obtained for all knowledge-related items. Higher scores suggested better HIV-related knowledge.

Students' attitudes towards PLHIV were also scored. One point was assigned for having negative feelings; two points were assigned for feeling indecisive i.e., not sure what to think or feel; and three points were assigned for expressing positive attitudes and support to PLHIV. The Attitude Score (ATS) was obtained by summing the assigned points per item. Higher scores represented a stronger positive attitude towards PLHIV.

### Main outcome measures

Participants were asked whether or not they had ever been tested for HIV (yes vs. no). We also evaluated their testing experiences and potential reasons for not having been tested (possible answer options: no, due to fear / no, due to finances / no need to get tested / does not know where to get tested).

Awareness and interest in HIV testing were assessed by asking the students about their position in case they wanted to take the test (possible answers: I know where to take the test / I do not know where to take the test, but I know whom to ask / the test cannot be taken in our country / I do not know, I do not care). Circling one of former two answers was classified as positive approach to HIV testing. Circling one of latter two answers was classified as negative approach to HIV testing.

### Statistical analysis

All analyses were performed in SPSS, version 21. Differences were tested using the ANOVA (F) and Kruskal-Wallis test (KW χ2). Spearman's correlation was used to investigate the associations between students' characteristics and different aspects of HIV testing.

**Supplemental Figure S1:**
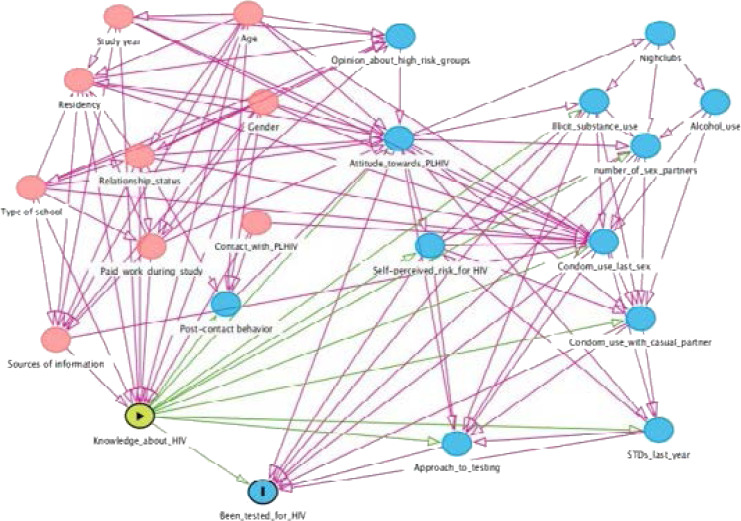
Initial DAG model of the association between knowledge about HIV and being tested for HIV

To analyse association between the variables, we used digital acyclic graphs (DAG) using an open-source platform www.dagitty.net. In DAG we considered “Knowledge about HIV” as the exposure was and “Been tested for HIV” as the outcome. We observed that the independent variables accounting for the socio-demographic characteristics of students were the main confounders, while the independent variables accounting for health behaviour were mediators of this association (Supplemental Figure S1). Therefore, a binary logistic regression was performed to evaluate factors associated with having been tested for HIV (yes/no) and having a positive vs. negative approach to HIV testing (dependent variables). All models included KNS and ATS. The independent variables were classified in five models based on the questionnaire sections: 1) Socio-demographics; 2) Information sources; 3) Awareness and interest in HIV testing; 4) Mindsets and 5) Lifestyle.

## Results

### Description of the study sample

Of 1,017 participants, 44.6% were males. The mean ± standard deviation (SD) age of students was 21.34 ± 3.51 years. One third (30.3%) studied health-related disciplines, and the majority (62.3%) were in the first study year. The internet was the main source of information about HIV (81.4%). The students generally visited nightclubs on weekends (58.3%), hardly ever used illicit drugs (4.6%), drank alcohol in moderation (64.7%) and had one or less sexual partners in the past year (74%). Approximately one third of students never used a condom. Study participants more often expressed a negative opinion (i.e., desire to protect themselves or avoid contact) when it comes to drug users (64.0%) and gays / lesbians (50.0%), while a smaller percentage of participants had a negative opinion regarding sex workers (42.4%) (Table 2).

**Table 2:** Attitudes towards HIV testing, high-risk groups for acquiring HIV and HIV information sources

Parameters		Previous HIV testing				Approach to HIV testing			
		Tested		Not tested		Tested		Not tested	
		N	%	N	%	N	%	N	%
Approach to testing	positive	53	5.2	668	65.7	/	/	/	/
	negative	2	0.2	294	28.9	/	/	/	/
Self-assessment of risk for HIV	very high	3	0.3	32	3.1	22	2.2	13	1.3
	high	4	0.4	47	4.6	38	3.7	13	1.3
	do not know	9	0.9	185	18.2	114	11.2	80	7.9
	low	22	2.2	229	22.5	196	19.3	55	5.4
	very low	17	1.7	469	46.1	351	34.5	135	13.3
Contact with PLHIV	had contact	12	1.2	25	2.5	33	3.2	4	0.4
	no verified	43	4.2	937	92.1	688	67.6	292	28.7
Actions after contact with PLHIV	not sure	14	1.4	194	19.1	152	14.9	56	5.5
	stop contact	1	0.1	97	9.5	54	5.3	44	4.3
	less contact	8	0.8	269	26.5	186	18.3	91	8.9
	same contact	32	3.1	402	39.5	329	32.4	105	10.3
Media information	yes	40	3.9	638	62.7	508	50.0	170	16.7
	no	15	1.5	325	32.0	213	20.9	126	12.4
Internet information	yes	47	4.6	779	76.6	613	60.3	215	21.1
	no	6	0.6	183	18.0	108	10.6	81	8.0
Medical information	yes	34	3.3	610	60.0	460	45.2	184	18.1
	no	21	2.1	352	34.6	261	25.7	112	11.0
Information univ. lecturers	yes	30	2.9	482	47.4	376	37.0	136	13.4
	no	25	2.5	480	47.2	345	33.9	160	15.7
Friends information	yes	34	3.3	604	59.4	489	48.1	149	14.7
	no	21	2.1	358	35.2	232	22.8	147	14.5
Special HIV education	yes	25	2.5	272	26.7	231	22.7	66	6.5
	no	30	2.9	690	67.8	490	48.2	230	22.6
Opinion on injecting drug users	their right	12	1.2	220	21.6	173	17.0	59	5.8
	avoiding contact	13	1.3	306	30.1	228	22.4	91	8.9
	need protection	23	2.3	309	30.4	235	23.1	97	9.5
	does not think	7	0.7	127	12.5	85	8.4	49	4.8
Opinion on gays/lesbians	their right	14	1.4	305	30.0	255	25.1	64	6.3
	avoiding contact	20	2.0	295	29.0	214	21.0	101	9.9
	need protection	11	1.1	182	17.9	135	13.3	58	5.7
	does not think	10	1.0	180	17.7	117	11.5	73	7.2
Opinion on sex workers	their right	25	2.5	310	30.5	251	24.7	84	8.3
	avoiding contact	12	1.2	254	25.0	189	18.6	77	7.6
	need protection	6	0.6	159	15.6	110	10.8	55	5.4
	does not think	12	1.2	239	23.5	171	16.8	80	7.9

Legend: PLHIV – persons living with HIV; univ – university.

### Approach and compliance with HIV testing

A positive approach to HIV testing was registered in 70.9% of the respondents. However, only 5.4% of students have taken the HIV test. Of 721 students who had a positive testing approach, only 53 (7.3%) had taken the test. Almost all (99.3%) students who had a negative testing approach had never taken the HIV test. Among the students who had not previously been tested, 69.4% were well aware of the testing facilities (Table 2).

The least frequent reason for non-compliance to HIV testing was financial constraint (0.7%). Fear of testing or receiving the test results prevented 54 (5.3%) students from testing. Three quarters (762, 74.9%) of students believed that they did not need to be tested. In addition, 266 (26.2%) were not interested in testing. Some students (139, 13.7%) did not know where to take the test, while 30 (2.9%) thought that HIV testing was not organized in the Serbitheir an Kosovo province.

Most students had adequate knowledge about HIV and expressed a positive attitude towards PLHIV. The mean ± SD KNS of students tested for HIV was 33.42 ± 3.50, while KNS of students who were never tested was 32.75 ± 3.21 (p = 0.12). Contrary, KNS of students who had a positive approach to testing was significantly higher than among those who had a negative approach to testing (tested mean ± SD ATS = 33.04 ± 3.10; not tested = 31.91 ± 3.58; p < 0.01). However, there were no significant differences (p = 0.43) between the tested and non-tested students relative to the ATS (tested students mean ± SD ATS = 39.50 ± 4.55; not tested = 39.06 ± 4.48). On the other hand, ATS of students who had a positive approach to testing was significantly higher compared to those students who had a negative approach (positive mean ± SD ATS = 39.81 ± 4.20; negative = 37.35 ± 4.63; p < 0.01). Students' characteristics, behaviours, mindsets and attitudes are presented in Table 2. Correlations and differences between the assessed variables and various aspects of HIV testing and approach to testing are presented in Supplemental Tables S1 and S2.

**Supplemental Table S1:** Correlations and differences of students' socio-demographic data relative to previous HIV testing, testing experience, attitude and approach

Parameters	Correlations (rho / p)				Differences (KW χ^2^ / p)			
	Tested for HIV before	HIV testing experience and reason	Interest in HIV testing	Approach to testing	Tested for HIV before	HIV testing experience and reason	Interest in HIV testing	Approach to testing
Has been tested for HIV before	/	**0.516**	**0.244**	**0.134**	/	**270.462**	**77.884**	**0.134**
	/	**0.001**	**0.001**	**0.001**	/	**0.001**	**0.000**	**0.001**
Previous HIV test experience	**0.516**	/	0.022	0.005	**270.462**	/	**21.129**	**18.261**
	**0.001**	/	0.487	0.866	**0.001**	/	**0.001**	0.001
Interest in HIV testing	**0.244**	0.022	/	**0.833**	**60.285**	**89.449**	/	**74.626**
	**0.001**	0.487	/	**0.001**	**0.001**	**0.001**	/	**0.001**
Approach to Testing	**0.134**	0.005	**0.833**	/	**18.261**	**26.950**	**116.000**	/
	**0.001**	0.866	**0.001**	/	**0.001**	**0.001**	**0.001**	/
Students gender	**0.100**	**0.149**	0.026	-0.039	**10.182**	**23.610**	**12.665**	1.513
	**0.001**	**0.001**	0.413	0.219	**0.001**	**0.001**	**0.005**	0.219
Students age	**0.102**	**0.120**	**0.209**	**0.157**	**10.567**	**21.575**	**45.743**	**25.076**
	**0.001**	**0.001**	**0.001**	**0.001**	**0.001**	**0.001**	**0.001**	**0.001**
School group (health/others)	0.013	-0.016	0.041	0.008	0.164	1.696	6.402	0.061
	0.686	0.607	0.193	0.805	0.685	0.791	0.094	0.805
School year	0.056	**0.069**	**0.165**	**0.105**	3.233	15.700	**31.845**	**11.173**
	0.072	**0.027**	**0.001**	**0.001**	0.072	0.003	**0.001**	**0.001**
Doing paid work	**-0.164**	**-0.091**	**-0.076**	0.029	**27.268**	**29.885**	**10.348**	0.877
	**0.001**	**0.004**	**0.015**	0.349	**0.001**	**0.001**	**0.016**	0.349
Relationship status	-0.012	0.014	**-0.072**	-0.038	**21.554**	**28.823**	6.956	1.446
	0.121	0.645	**0.022**	0.229	**0.001**	**0.004**	0.073	0.229
Accommodation during schooling	-0.042	-0.003	0.014	0.044	1.808	2.383	3.753	1.942
	0.179	0.911	0.660	0.164	0.179	0.666	0.289	0.163
Knowledge score	-0.058	-0.005	**0.167**	**0.156**	3.373	4.238	**29.293**	**24.779**
	0.066	0.871	**0.001**	**0.001**	0.066	0.375	**0.001**	**0.001**
Attitude score	-0.028	0.007	**0.255**	**0.260**	0.775	1.786	**75.441**	**68.764**
	0.379	0.830	**0.001**	**0.001**	0.379	0.775	**0.001**	**0.001**

**Supplemental Table S2:** Correlations and differences in information sources, mindsets and lifestyle relative to previous HIV testing, testing experience, interest and approach to HIV testing

Parameters	Correlations (rho / p)				Differences (KW χ 2 / p)			
	Tested for HIV before	HIV testing experience and reason	Interest in HIV testing	Approach to testing	Tested for HIV before	HIV testing experience and reason	Interest in HIV testing	Approach to testing
Self- assessment of risk HIV inf.	**-0.063**	**-0.179**	**-0.065**	**-0.064**	**3.995**	**36.457**	5.713	**4.140**
	**0.046**	**0.001**	**0.037**	**0.042**	**0.046**	**0.001**	0.126	**0.042**
Previous contact with PLHIV	**0.232**	**0.100**	**0.127**	**0.078**	**54.763**	**59.098**	**18.960**	**6.222**
	**0.001**	**0.001**	**0.001**	**0.013**	**0.001**	**0.001**	**0.000**	**0.013**
Actions after contacting PLHIV	-0.045	0.036	**0.091**	**0.070**	2.101	7.410	**8.475**	**4.939**
	0.147	0.252	**0.004**	**0.026**	0.147	0.116	**0.037**	**0.026**
Opinion on inject drug users	-0.026	-0.009	**0.086**	**0.062**	0.663	0.895	7.795	**3.893**
	0.416	0.775	**0.006**	**0.048**	0.415	0.925	0.050	**0.048**
Opinion on gays/lesbians	-0.016	-0.030	**0.143**	**0.134**	0.266	2.418	**21.522**	**18.252**
	0.606	0.338	**0.001**	**0.001**	0.606	0.659	**0.001**	**0.001**
Opinion on sex workers	0.052	**0.079**	**0.120**	**0.065**	2.721	7.065	**21.131**	**4.307**
	0.099	**0.012**	**0.001**	**0.038**	0.099	0.132	**0.001**	**0.038**
Nightclubs	-0.033	-0.008	0.008	-0.010	**8.408**	3.934	4.245	0.093
	0.289	0.801	0.806	0.760	**0.038**	0.415	0.236	0.760
Alcohol intake	0.015	0.047	0.037	0.046	0.225	3.645	2.278	2.141
	0.636	0.131	0.238	0.143	0.636	0.456	0.517	0.143
Opioids (drugs) use	0.051	**0.085**	-0.005	-0.003	2.632	**9.947**	0.162	0.011
	0.105	**0.007**	0.879	0.916	0.105	**0.041**	0.983	0.916
Number of sex partners last year	**0.096**	**0.170**	0.029	0.020	**9.317**	**31.416**	4.625	0.421
	**0.002**	**0.001**	0.361	0.517	**0.002**	**0.001**	0.201	0.516
Condom use at last sex	-0.015	0.012	**0.078**	**0.072**	0.234	1.302	6.347	**5.231**
	0.629	0.709	0.013	**0.022**	0.628	0.861	0.096	**0.022**
Condom use with new partner	0.016	**0.090**	**0.071**	0.037	**13.863**	**9.542**	6.986	1.368
	0.601	**0.004**	**0.023**	0.242	**0.008**	**0.049**	0.072	0.242
STDs in last year	0.007	0.023	-0.053	-0.033	0.046	**14.225**	3.293	1.127
	0.831	0.460	0.089	0.289	0.831	**0.007**	0.349	0.288
Media sources for information	0.031	0.032	**0.077**	**0.126**	0.960	3.971	**20.428**	**16.005**
	0.327	0.302	**0.014**	**0.000**	0.327	0.410	**0.001**	**0.001**
Internet sources for information	0.047	-0.022	**0.108**	**0.145**	2.264	**12.209**	**23.306**	**21.255**
	0.133	0.492	**0.001**	**0.001**	0.087	**0.016**	**0.001**	**0.001**
Medical sources for information	-0.007	-0.006	0.025	0.015	0.057	0.265	2.152	0.242
	0.812	0.840	0.422	0.623	0.812	0.992	0.542	0.623
Univ. lecturers for information	0.020	0.002	0.053	0.056	0.410	1.022	3.367	3.228
	0.522	0.951	0.089	0.072	0.522	0.906	0.338	0.072
Information from friends	-0.005	0.042	**0.089**	**0.164**	0.021	4.265	**35.249**	**27.414**
	0.885	0.186	**0.005**	**0.000**	0.885	0.371	**0.001**	**0.001**
Receiving special education on HIV	**0.085**	0.017	**0.100**	**0.097**	**7.427**	**11.432**	**10.969**	**9.622**
	**0.006**	0.586	**0.001**	**0.002**	**0.006**	**0.022**	**0.012**	**0.002**

### Regression models

Logistic regression examining compliance with HIV testing revealed significant equations (B = 2.86; Wald = 426.04; Exp(B) = 17.49) for two variable groups (Socio-demographic χ2 = 41.45; p < 0.01 and Awareness and interest in HIV testing χ2 = 91.33; p < 0.01). The Information sources (χ2 = 12.36; p = 0.13), Mindsets (χ2 = 7.99; p = 0.15) and Lifestyle (χ2 = 15.76; p = 0.07) models were not significant (Table 3). Factors associated with previous HIV testing were being male and younger, having interest in HIV testing and having previous contact with PLHIV.

**Table 3:** Binary logistic regression models examining factors associated with previous HIV testing

Tested for HIV before (yes/no)		B	Wald	p	OR	Cl Low	Cl High
Model 1. Socio- demographic R_2_ Nagelkerke=0.116 classification=94.6%	KN score	-0.074	2.375	0.123	0.929	0.845	1.020
	AT score	0.007	0.036	0.849	1.007	0.940	1.078
	**Sex**	0.790	6.757	**0.009**	2.203	1.215	3.998
	**Age**	-0.134	12.393	**0.001**	0.874	0.811	0.942
	School group	0.131	0.170	0.680	1.140	0.612	2.123
	School year	0.117	0.955	0.329	1.124	0.889	1.422
	Doing paid work	0.719	2.288	0.130	2.052	0.809	5.207
	Relation status	0.159	0.268	0.605	1.172	0.643	2.136
	Accommodation	-0.047	0.124	0.725	0.954	0.734	1.240
Model 2. Information sources	KN score	-0.057	1.335	0.248	0.945	0.859	1.040
	AT score	-0.003	0.005	0.942	0.997	0.931	1.068
	Media sources	0.141	0.169	0.681	1.152	0.587	2.260
	Internet sources	0.612	1.619	0.203	1.844	0.719	4.730
	Medical sources	-0.103	0.113	0.736	0.902	0.496	1.641
	University lecturers	-0.033	0.011	0.915	0.968	0.529	1.771
	Friends as source	-0.324	1.068	0.301	0.723	0.391	1.338
	Special education	0.749	7.142	**0.008**	2.114	1.221	3.660
Model 3. Awareness and interest in HIV testing R_2_ Nagelkerke=0.251 classification=94.7%	KN score	-0.060	1.337	0.248	0.942	0.851	1.042
	AT score	0.031	0.676	0.411	1.032	0.958	1.111
	**Interest in testing**	1.802	24.544	**0.001**	6.059	2.971	12.358
	Self-risk assessment	0.230	3.169	0.075	1.258	0.977	1.621
	**Contact with PLHIV**	1.866	19.522	**0.001**	6.460	2.824	14.778
	Post-contact action	-0.016	0.014	0.904	0.984	0.755	1.282
Model 4. Mindsets	KN score	-0.061	1.581	0.209	0.941	0.856	1.034
	AT score	-0.011	0.097	0.756	0.989	0.924	1.059
	Opinion injects drug users	-0.199	1.394	0.238	0.820	0.589	1.140
	Opinion gays/lesbians	-0.156	1.016	0.314	0.856	0.632	1.159
	Opinion sex workers	0.320	4.721	**0.030**	1.377	1.032	1.837
Model 5. Lifestyle	KN score	-0.070	2.050	0.152	0.933	0.848	1.026
	AT score	-0.010	0.077	0.781	0.990	0.925	1.060
	Nightclubs	-0.332	2.296	0.130	0.717	0.467	1.102
	Alcohol use	0.030	0.010	0.921	1.030	0.576	1.842
	Opioids (drugs) use	0.628	1.452	0.228	1.874	0.675	5.203
	Sex partners number	0.997	9.287	0.002	2.711	1.428	5.150
	Condom in last sex	-0.193	0.397	0.528	0.825	0.453	1.502
	Condom casual partner	-0.032	0.106	0.745	0.968	0.797	1.176
	Sex transmitted disease	-0.052	0.005	0.945	0.949	0.212	40.242

Legend: KN – knowledge; AT – attitudes; PLHIV – persons living with HIV; B-unstandardized coefficient, OR – odds ratio, CI – confidence interval; p – probability level; bolded values are statistically significant

Logistic regression examining positive approach to HIV testing revealed significant equations (B = -0.89; Wald = 166.32; Exp(B) = 0.411) for all the variable groups (Socio-demographic χ2 = 91.76; p < 0.01; Information sources χ2 = 112.78; p < 0.01; Awareness and interest in HIV testing χ2 = 81.49; p < 0.01; Mindsets χ2 = 82.46; p < 0.01; Lifestyle χ2 = 82.79; p < 0.01) (Table 4). Factors associated with positive approach to HIV testing were having higher knowledge about HIV and stronger positive attitude towards PLHIV, being older, having received information about HIV through friends and special educational programs, using condom at last sex, having positive opinion on gays/lesbians and previous contact with PLHIV.

**Table 4:** Logistic regression models examining factors associated with positive approach to HIV testing

Approach to testing (positive/negative)		B	Wald	p	OR	Cl Low	Cl High
Model 1. Sociodemographic R^2^ Nagelkerke=0.123 classification=71.1%	**KN score**	-0.050	4.301	**0.038**	0.951	0.907	0.997
	**AT score**	-0.111	37.207	**0.001**	0.895	0.863	0.927
	Sex	-0.161	1.152	0.283	0.852	0.635	1.142
	**Age**	-0.109	6.248	**0.012**	0.897	0.823	0.977
	School group	0.071	0.188	0.665	1.073	0.780	1.477
	School year	0.029	0.151	0.698	1.029	0.890	1.190
	Doing paid work	0.048	0.016	0.900	1.049	0.500	2.199
	Relation status	0.043	0.149	0.700	1.044	0.839	1.300
	Accommodation	0.117	3.571	0.059	1.124	0.996	1.269
Model 2. Information sources R^2^ Nagelkerke=0.151; classification=81.5%	KN score	-0.044	3.203	0.073	0.957	0.912	1.004
	**AT score**	-0.118	41.584	**0.001**	0.889	0.858	0.921
	Media sources	0.271	2.500	0.114	1.312	0.937	1.836
	Internet sources	0.369	3.497	0.061	1.447	0.982	2.130
	Medical sources	0.140	0.757	0.384	1.150	0.840	1.575
	University lecturers	-0.154	0.921	0.337	0.858	0.627	1.174
	**Friends as source**	0.555	11.685	**0.001**	1.743	1.267	2.396
	**Special education**	0.404	5.235	**0.022**	1.499	1.060	2.119
Model 3. Awareness and interest in HIV testing R^2^ Nagelkerke=0.111; classification=71.8%	**KN score**	-0.047	3.862	**0.049**	0.954	0.910	1.000
	**AT score**	-0.118	41.528	**0.001**	0.889	0.858	0.921
	Tested for HIV before	0.053	0.706	0.401	1.055	0.932	1.194
	Self-risk assessment	-0.059	0.782	0.376	0.943	0.828	1.074
	**Contact with PLHIV**	1.186	4.575	**0.032**	3.274	1.104	9.708
	Post-contact action	0.019	0.089	0.765	1.020	0.897	1.159
Model 4. Mindsets R2 Nagelkerke=0.111; classification=71.6%	**KN score**	-0.047	3.868	**0.049**	0.954	0.910	1.000
	**AT score**	-0.113	39.438	**0.001**	0.893	0.862	0.925
	Opinion injects drug users	0.019	0.050	0.823	1.020	0.860	1.209
	**Opinion gays/lesbians**	0.191	5.588	**0.018**	1.211	1.033	1.419
	Opinion sex workers	-0.006	0.008	0.930	0.994	0.860	1.148
Model 5. Lifestyle R^2^ Nagelkerke=0.112; classification=71.5%	**KN score**	-0.048	4.042	**0.044**	0.953	0.909	0.999
	**AT score**	-0.121	44.388	**0.001**	0.886	0.855	0.918
	Nightclubs	-0.133	1.390	0.238	0.875	0.701	1.092
	Alcohol use	0.210	1.907	0.167	1.234	0.916	1.662
	Opioids (drugs) use	0.134	0.147	0.701	1.144	0.576	2.273
	Sex partners number	0.077	0.177	0.674	1.080	0.754	1.548
	**Condom in last sex**	0.347	4.787	**0.029**	1.415	1.037	1.931
	Condom casual partner	-0.019	0.150	0.698	0.981	0.893	1.079
	Sex transmitted disease	-0.222	0.299	0.585	0.801	0.362	10.773

Legend: KN – knowledge; AT – attitudes; PLHIV – persons living with HIV; B-unstandardized coefficient, OR – odds ratio, CI – confidence interval; p – probability level; bolded values are statistically significant.

## Discussion

### Principal findings

This study found that a very small proportion of students had previously been tested for HIV, even though the majority had positive approach to HIV testing. Having better knowledge about HIV and positive attitudes towards PLHIV was associated with having positive approach to HIV testing, but not with previous HIV testing. The results of this study suggest that there is a difference in factors associated with previous HIV testing and having a positive approach to HIV testing.

### Comparison with similar studies

Previous studies suggested that a lower level of HIV-related knowledge is often coupled with HIV-related stigma when deciding whether or not to take the test 14. Fear of stigmatization is a major barrier to HIV testing 3,15,16. This is commonly related to social labelling, such as being an injecting drug user, sex worker, gay/lesbian, or already being HIV positive.

A review of literature conducted in low- and middle-income countries found that adequate HIV-related knowledge decreases the HIV-related stigma, which in turn increases the rates of HIV testing 17. However, studies conducted in the United States and South African Republic suggested that higher knowledge about HIV did not influence the intention to take the test 18,19.

Feelings of fear and a sense of invulnerability have also been identified as major emotional barriers to HIV testing in college students 15. Interestingly, only 5.3% of our students reported fear as a reason for non-compliance to HIV testing. This stands in contrast with the results of other studies 15,19. In a previous study of high school pupils in Serbia, fear was found to be one of the most common reasons for non-compliance, despite having a positive attitude and being willing to take the test.

About one-third of students experience less stigmatization in the academic setting compared to their home environment^20^ and a greater availability of HIV counselling and testing services facilitates compliance with HIV testing^15^,^16^. Inconsistent condom use and practice of other risky behaviours have been associated with higher HIV testing rates^21^. Also, persons who do not use condoms consistently and frequently changes sexual partners are more likely to be aware of a higher HIV risk, which, in turn, supports a higher testing compliance^22^,^23^.

Health education programs, especially peer education, had a positive impact on one's approach to HIV testing^24^,^25^. The advantages of peer education come from the fact that the audience can relate to their educators who use a similar language and do not have an authoritarian role. The problem of HIV testing could be addressed by bridging the cognitive (knowing that testing is useful and where and how it could be done) and emotional (fear of the consequences of testing and feeling invulnerable to HIV infection) dimensions.

### Importance of the study for public health

The findings from this study suggest that knowledge and attitudes towards PLHIV go hand-in-hand, and therefore, peer education programs aiming at increasing HIV-related knowledge should address attitudes and treatment of PLHIV as well. Our results add to the body of evidence that being more knowledgeable about HIV was not associated with a previous HIV testing experience among university students living in a high-risk region for HIV transmission. The finding that most students declared that they did not need to take the test supports the assumption that most students in this study were at a low risk of HIV exposure (never using drugs, not being sexually active, consistent condom use and not having multiple sexual partners) while being knowledgeable about HIV.

Based on the results from this study, the contact with PLHIV could help reduce fear of HIV and HIV-related stigma, and therefore, facilitate the increase in HIV testing uptake. We observed that special education about HIV was associated with having a positive approach to HIV testing, and that interest in HIV testing was associated with previous HIV testing. Involvement of PLHIV in peer education on HIV could help to increase the awareness of HIV testing among young adults.

A total of 13.7% of students were misinformed that HIV testing is not available in their country even though HIV testing in Kosovo has been free of charge for many years. A lack of facilities offering confidential counselling and HIV testing in the northern Kosovo province could explain the low HIV testing rates in this study. Specifically, Students' Public Health Centers, where students are obliged to come for periodic health checks, are sui

 venues in which students could receive adequate HIV-related information and change their perception of HIV testing.

### Cues to action

Low testing rates in students may delay timely diagnosis and treatment. This facilitates the transmission of HIV infection as the incidence of HIV in young people rises 26,27. Therefore, it is essential to enhance the rate of testing. First, accessibility of counselling can strongly influence the change in health-related behaviours that affect the HIV risk reduction 28. In more urbanized settings calls for testing could be broadcasted through mass media and Internet (short video clips, banners etc.), while people who work with PLHIV (such as health professionals, counsellors, etc.) should publicly discuss the relevance of testing and their experiences from the counselling center. In less urbanized settings, the efforts to increase the HIV testing rates should focus on the trusted members of the local community, such as local health professionals, athletes, artists, academics or representatives of the religious communities, who may publicly discuss the issues surrounding HIV.

The channels of communication with young people have evolved along with the technological advancement 29–31. Previous studies indicated that interventions based on the use of new technologies can lead to an increase in HIV testing among young people 29. In this way users are able to estimate individual risks by filling in a simple online test or understand the risks through video materials or computer games 29–31. Also, this approach helps to overcome barriers to HIV testing in young people such as stigma and discrimination, low perception of personal risk, presence and monitoring of parents, lack of privacy and confidentiality 29^–32^. Use of digital platforms allows for an easier access to counselling. Furthermore, banners, popup windows and reminders about prevention of HIV and testing should be included in dating and socializing apps. Finally, the HIV testing is also related to the availability of the testing kits. To increase the testing rate, it is essential to ensure the availability of HIV tests 29, 30.

## Study limitations

This study explored a sensitive topic, and it is, therefore, open to information bias, because all information about students including previous testing experience were self-reported. Due to a cross-sectional study design, the direction of the association cannot be defined. Therefore, the inference on causal pathways can be limited. This study was an integral part of a larger research about knowledge, attitudes and behaviours related to HIV. Because this study was part of a PhD project, the entire investigation had to be completed before the results could be published, which may constitute a limitation due to delayed reporting. Nevertheless, because the overall social and political circumstances in Kosovo have not markedly changed since the end of the armed conflict in 1999, the obtained results are still novel and relevant. Specifically, policies with regards to sex education have remained similar to that when the study was conducted i.e. sex education has not yet been part of the school program. For this reason, knowledge about HIV and attitudes towards PLHIV observed in this study reflect the current state of affairs.

## Conclusion

In conclusion, we report a discrepancy between a large proportion of students who had a positive approach to testing and a small proportion of students who have previously taken the HIV test. Having more knowledge about HIV and positive attitude about PLHIV was associated with having a positive approach to HIV testing, but not with compliance to testing. Given the overall positive approach to HIV testing, access to testing facilities to increase the HIV testing rates should be one of public health priorities in the province. Promotion of education about HIV and contact with HIV positive persons might play an important role in forming positive approach to HIV testing.

## Figures and Tables

**Table 1:** Socio-demographic characteristics and HIV-related behaviours among students

Parameters		Previous HIV testing				Approach to HIV testing			
		Tested		Not tested		Positive		Negative	
		N	%	N	%	N	%	N	%
Sex	male	36	3.5	418	41.1	313	30.8	141	13.9
	female	19	1.9	544	53.5	408	40.1	155	15.2
School groups	biomedical	18	1.8	290	28.5	220	21.6	88	8.7
	others	37	3.6	672	66.1	501	49.3	208	20.5
School year	first	28	2.8	606	59.6	426	41.9	208	20.5
	fourth	27	2.7	356	35.0	295	29.0	88	8.7
Doing paid work	yes	12	1.2	47	4.6	45	4.4	14	1.4
	no	43	4.2	915	90.0	676	66.5	282	27.7
Relationship status	single	24	2.4	446	43.9	326	32.1	144	14.2
	in a relation	19	1.9	458	45.0	341	33.5	136	13.4
	partner living	1	0.1	8	0.8	8	0.8	1	0.1
	married	11	1.1	50	4.9	46	4.5	15	1.5
Accommodation during schooling	dormitory	10	1.0	273	26.8	199	19.6	84	8.3
	rented house	17	1.7	294	28.9	237	23.3	74	7.3
	in own home	12	1.2	118	11.6	91	8.9	39	3.8
	with parents	16	1.6	227	22.3	194	19.1	99	9.7
Frequenting nightclubs	every night	3	0.3	32	3.1	19	1.9	16	1.6
	during week	10	1.0	154	15.1	120	11.8	44	4.3
	on weekends	23	2.3	577	56.7	428	42.1	172	16.9
	no clubbing	19	1.9	199	19.6	154	15.1	64	6.3
Alcohol intake	until drunk	5	0.5	60	5.9	37	3.6	28	2.8
	moderately	35	3.4	628	61.8	497	48.9	166	16.3
	does not drink	15	1.5	274	26.9	187	18.4	102	10.0
Opioid use	yes	5	0.5	42	4.1	33	3.2	14	1.4
	no	50	4.9	920	90.5	688	67.6	282	27.7
Sex partners during past year	more than one	24	2.4	241	23.7	192	18.9	73	7.2
	one or less	31	3.0	721	70.9	529	52.0	223	21.9
Condom use with a casual partner	always	23	2.3	372	36.6	296	29.1	99	9.7
	sometimes	5	0.5	175	17.2	112	11.0	68	6.7
	only then	9	0.9	58	5.7	51	5.0	16	1.6
	never	5	0.5	48	4.7	33	3.2	20	2.0
	no sex	13	1.3	309	30.4	229	22.5	93	9.1
Condom use at last sex	yes	24	2.4	542	53.3	354	34.8	122	12.0
	no	31	3.0	510	50.1	367	36.1	174	17.1
STDs in past year	had	2	0.2	30	2.9	20	2.0	12	1.2
	did not have	53	5.2	932	91.6	701	68.9	284	27.9

Legend: STD – sexually transmitted disease; KN – knowledge; AT – attitude.

## References

[1] Institute of Public Health of Serbia (2014). Results of the national health survey of Serbia, 2013.

[2] Duric P, Ilic S, Rajcevic S (2013). Provider-initiated vs. client-initiated HIV testing in Autonomous Province of Vojvodina, Serbia, 2000–2008. Journal of Infection in Developing Countries.

[3] Delva W, Wuillaume F, Vansteelandt S, Claeys P, Verstraelen H, Vanden Broeck D, Temmerman M (2008). HIV testing and sexually transmitted infection care among sexually active youth in the Balkans. AIDS Patient Care STDS.

[4] The Government of the Republic of Serbia (2018). National Strategy for the Prevention and Control of HIV/AIDS in the Republic of Serbia, 2018-2025. Official Gazette of the Republic of Serbia no. 61/2018.

[5] Institute of Public Health of Serbia (2019). Annual Report on Communicable Diseases.

[6] Levi J, Raymond A, Pozniak A, Vernazza P, Kohler P, Hill A (2016). Can the UNAIDS 90-90-90 target be achieved? A systematic analysis of national HIV treatment cascades. BMJ Glob Health.

[7] Prabhu S, Harwell JI, Kumarasamy N (2019). Advanced HIV: diagnosis, treatment, and prevention. Lancet HIV.

[8] Cousins S (2018). HIV in Serbia: stigma and a stagnant HIV response. Lancet HIV.

[9] World Health Organization (WHO) (2020). Review of the HIV programme in Kosovo (in accordance with United Nations Security Council Resolution 1244 (1999)).

[10] Romanian Harm Reduction Network (2006). UN administered province of Kosovo, Most-at-risk adolescents and young people, HIV and substance use. Country Mission Report.

[11] Institute for Students' Health Care (2020). Department for Healthcare promotion and prevention.

[12] UNAIDS – Joint United Nations Program on HIV/AIDS (2015). Republic of Serbia; Reporting period: January-December 2014.

[13] Meil WM, LaPorte DJ, Mills JA, Sesti A, Collins SM, Stiver AG (2016). Sensation seeking and executive deficits in relation to alcohol, tobacco, and marijuana use frequency among university students: Value of ecologically based measures. Addictive Behaviors.

[14] Ijeoma A, Ejikeme A, Theodora O, Chika O (2018). Knowledge, attitude, willingness of HIV counseling and testing and factors associated with it, among long distant drivers in Enugu, Nigeria: an opportunity in reduction of HIV prevalence. African Health Sciences.

[15] Lin CA, Roy D, Dam L, Coman EN (2017). College students and HIV testing: cognitive, emotional self-efficacy, motivational and communication factors. Journal of Community Healthcare.

[16] Gwadz M, Leonard NR, Honig S, Freeman R, Kutnick A, Ritchie AS (2018). Doing battle with “the monster:” how high-risk heterosexuals experience and successfully manage HIV stigma as a barrier to HIV testing. International Journal for Equity in Health.

[17] Thapa S, Hannes K, Cargo M, Buve A, Peters S, Dauphin S, Mathei C (2018). Stigma reduction in relation to HIV test uptake in low- and middle-income countries: a realist review. BMC Public Health.

[18] Deblonde J, De Koker P, Hamers FF, Fontaine J, Luchters S, Temmerman M (2010). Barriers to HIV testing in Europe: a systematic review. European Journal of Public Health.

[19] Chunloy K, Apisarnthanarak A (2016). Uptake of HIV testing and counseling, risk perception and linkage to HIV care among Thai university students. BMC Public Health.

[20] Haffejee F, Maughan-Brown B, Buthelezi T, Kharsany ABM (2018). Perceived HIV-related stigma among university students in South Africa: implications for HIV testing. African Journal of AIDS Research.

[21] Xu H, Xie J, Xiao Z, Li X, Goldsamt L, Bartley Williams A, Wang H (2019). Sexual attitudes, sexual behaviors, and use of HIV prevention services among male undergraduate students in Hunan, China: a cross-sectional survey. BMC Public Health.

[22] Rehm J, Shield KD, Joharchi N, Shuper PA (2012). Alcohol consumption and the intention to engage in unprotected sex: systematic review and meta-analysis of experimental studies. Addiction.

[23] Yi S, Tuot S, Chhoun P, Pal K, Chhim K, Ngin C, Brody C (2018). Sexual behaviors, HIV knowledge, HIV testing attitudes and recent HIV testing among female entertainment workers in Cambodia: A cross-sectional study. PLoS One.

[24] Menna T, Ali A, Worku A (2015). Effects of peer education intervention on HIV/AIDS related sexual behaviors of secondary school students in Addis Ababa, Ethiopia: a quasi-experimental study. Reprod Health.

[25] Tolli MV (2012). Effectiveness of peer education interventions for HIV prevention, adolescent pregnancy prevention and sexual health promotion for young people: a systematic review of European studies. Health Education Research.

[26] Dailey AF, Hoots BE, Hall H, Song R, Hayes D, Fulton P, Prejean J, Hernandez AL, Koenig LJ, Valleroy LA (2017). Vital signs: human immunodeficiency virus testing and diagnosis delays -United States. MMWR Morb Mortal Wkly Rep.

[27] Fauci AS, Redfield RR, Sigounas G, Weahkee MD, Giroir BP (2019). Ending the HIV epidemic: a plan for the United States. J Am Med Assoc.

[28] Dowshen N, Lee S, Matty Lehman B, Castillo M, Mollen C (2015). IknowUshould2: feasibility of a youth-driven social media campaign to promote STI and HIV testing among adolescents in Philadelphia. AIDS Behav.

[29] Ibitoye M, Lappen H, Freeman R, Jordan AE, Aronson ID (2021). Technology-Based Interventions to Increase Point-of-Care HIV Testing and Linkage to Care Among Youth in the US: A Systematic Review. AIDS Behav.

[30] Castel AD, Wilbourn B, Trexler C, D'Angelo LD, Greenberg D (2021). A Digital Gaming Intervention to Improve HIV Testing for Adolescents and Young Adults: Protocol for Development and a Pilot Randomized Controlled Trial. JMIR Res Protoc.

[31] Bumgarner KF, Pharr J, Buttner M, Ezeanolue E (2017). Interventions that increase the intention to seek voluntary HIV testing in young people: a review. AIDS Care.

